# All-organic electrostrictive polymer composites with low driving electrical voltages for micro-fluidic pump applications

**DOI:** 10.1038/srep11814

**Published:** 2015-07-03

**Authors:** Minh Quyen Le, Jean-Fabien Capsal, Jérémy Galineau, Florent Ganet, Xunqian Yin, Mingchia (Dawn) Yang, Jean-François Chateaux, Louis Renaud, Christophe Malhaire, Pierre-Jean Cottinet, Richard Liang

**Affiliations:** 1 Laboratoire de Génie Electrique et Ferroélectricité (LGEF), INSA Lyon, 69621 Villeurbanne, France; 2 Université de Lyon, Institut des Nanotechnologies de Lyon INL-UMR5270, CNRS, Université Lyon 1, Villeurbanne, F-69622, France; 3 Université de Lyon, Institut des Nanotechnologies de Lyon INL-UMR5270, CNRS, INSA de Lyon, 7 av. Jean Capelle, bât. Blaise Pascal, Villeurbanne, F-69621, France; 4 High-Performance Materials Institute (HPMI) Florida State University Materials Research Building 2005 Levy Ave, Tallahassee, FL 32310, USA

## Abstract

This paper focuses on the improvement of a relaxor ferroelectric terpolymer, i.e., poly (vinylidene fluoride-trifluoroethylene-chlorofluoroethylene) [P(VDF-TrFE-CFE)], filled with a bis(2-ethylhexyl) phthalate (DEHP). The developed material gave rise to a significantly increased longitudinal electrostrictive strain, as well as an increased mechanical energy density under a relatively low electric field. These features were attributed to the considerably enhanced dielectric permittivity and a decreased Young modulus as a result of the introduction of only small DEHP plasticizer molecules. In addition, the plasticizer-filled terpolymer only exhibited a slight decrease of the dielectric breakdown strength, which was a great advantage with respect to the traditional polymer-based electrostrictive composites. More importantly, the approach proposed herein is promising for the future development and scale-up of new high-performance electrostrictive dielectrics under low applied electrical fields through modification simply by blending with a low-cost plasticizer. An experimental demonstration based on a flexible micro-fluidic application is described at the end of this paper, confirming the attractive characteristics of the proposed materials as well as the feasibility of integrating them as micro-actuators in small-scale devices.

Actuators are controllable work-producing devices, based on various physical principals, exhibiting a wide array of performance capabilities^1^,^2^,^3^. The choice of a particular material for an actuator application depends on several performance metrics^1^. Electroactive polymers (EAPs) are materials capable of realizing energy conversion between electrical and mechanical energy, and have attracted great attention from researchers during the past decades for their potential applications in the fields of artificial muscles, actuators and energy harvesting^3^,^4^. Furthermore, EAPs possess numerous advantages such as low densities, mechanical flexibility, ease of processing, excellent electrical insulation properties and low cost, making them good candidates for practical applications^5^,^6^.

Among all EAPs, ferroelectric PVDF, its copolymers poly(vinylidene fluoride-co-trifluoroethylene) [P(VDF-TrFE)], and the terpolymer poly(vinylidene fluoride-trifluoroethylene-chlorofluoroethylene) [P(VDF-TrFE-CFE)] have been intensively investigated due to their very high electromechanical and electrocaloric responses^7^. Table 1 presents the electromechanical properties of such ferroelectric polymers. However, a requirement is currently unmet in actuation technologies: actuators do not provide the soft, smooth motions^2^,^3^ needed for mimicking the flow and pumping of blood in veins.

The property chart in Fig. 1 provides a comparison of electrostrictive polymers as well as other common actuators for two important actuation metrics including actuation stress and strain. As is known, smart materials like piezoceramics, magnetostrictive materials, shape memory alloys, etc. are usually used in the construction of low-strain actuators^11^,^12^. For instance, magnetostrictive or piezostrictive materials exhibit a typical actuation strain of only 10^−3^%^13^. Shape Memory Alloys, SMAs, on the other hand, allow significant actuation stress and strain but their typical working frequency is below 1 Hz^14^. Electrostrictive polymers, which can provide larger strain values and better dynamic properties as well as a sensing mechanism, are therefore interesting candidates for transducer applications.

However, most current actuation mechanisms depend on the electromagnetic devices, resulting in difficult fabrication at small length scales^11^. Typically, in magnetic MEMS devices, the maximum achievable displacement is between 10^−5^ and 10^−3^ m and the maximum force is between 10^−7^ and 10^−4^ N^15^. Hence, an existing challenge today is the development of electrostrictive systems with high mechanical energy density making it possible to attain greater work and force output at the microscale.

Electrostrictive polymers are attractive candidates for micro-actuator applications due to an easy manufacturing and a high mechanical energy density. Previous research has demonstrated a feasibility of employing an electrostrictive polymer as an actuator; showing that the actuation stress values of electrostrictive polymer surpasses the maximum sustainable stress values of mammalian skeletal muscles^8^,^16^. Recently, improvements in electrostrictive performance have significantly increased its competitiveness, especially among high electric field-driven actuators. It is clear from Fig. 1 that the main limitation of electrostrictive polymers is the level of actuation stress, which needs to be considerably improved, and, as well, the electric field in order to obtain large strains.

For dielectric polymers, the electrostrictive strain under an electric field can be mainly attributed to Maxwell forces induced by dipolar orientation within the material^17^,^18^. In the longitudinal direction, the compressive Maxwell strain and the mechanical energy density under the electric field are given by equations (1) and (2), respectively:


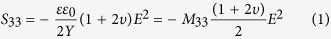



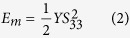


where *S*_33_ is the longitudinal strain along the thickness direction, *E*_*m*_ is the mechanical energy density, *ε* is the relative dielectric permittivity of the material, *ε*_0_ is the vacuum dielectric permittivity, *ν* is the Poisson ratio, *Y* is the Young modulus, *E* is the applied electric field and *M*_33_ is the longitudinal electrostrictive strain constant.

It can be easily deduced from equation (1) that the large electric fields required to achieve sufficient electrostrictive strains (a few percent) can be overcome by reducing the Young modulus of the polymer and/or increasing the dielectric permittivity. However, for low frequencies, typically below 10 Hz for actuator applications, the mechanical energy density, determined by equation (2), must also be enhanced. As a result, the increase in strain must be more significant than the decrease in the material’s elasticity modulus.

Recently, Capsal^16^ *et al.* proposed an easy solution to increase the electromechanical performances of a fluorinated terpolymer. The results showed that the doping of the polymer with a plasticizer led to an increase in molecular mobility of the macromolecule, inducing a decrease of the material’s Young modulus. Since many polymers are heterogeneous systems by nature, an increased molecular mobility enables - above the glass transition temperature and under an applied electric field - the trapping of charges at the boundaries of the heterogeneities within the polymer due to chain movement and morphological changes^19^.

In the case of semi-crystalline polymers, the charges trapped at the amorphous/crystalline phase boundaries induce large Maxwell-Wagner-Sillars polarization effects. This kind of interfacial polarization is associated with a significant increase of the dielectric permittivity at low frequency. Thus, choosing an adequate polymer matrix/plasticizer combination will enable the generation of large macroscopic dipoles associated with phase heterogeneities while reducing the Young modulus of the polymer, and thus increasing its dielectric permittivity. As a consequence, as shown in Equations (1) and (2), such phase engineering enhances both the electrostrictive strain and the generated mechanical energy density.

Here we first propose an all-organic polymer based on the P(VDF-TrFE-CFE) terpolymer matrix filled with the DEHP plasticizer to achieve an adequate heterogeneous morphology to enhance the actuation performance. Subsequently, such a material is applied in the development of a micro-fluidic pump.

Figure 2 shows DSC thermograms of the P(VDF-TrFE-CFE)/DEHP composites around their melting peaks. Each thermogram was normalized based on the mass of P(VDF-TrFE-CFE). As expected, it can be seen in Fig. 2 that the introduction of DEHP resulted in a significant linear decrease of the melting temperature from 129 °C for the pure terpolymer to 122 °C for a P(VDF-TrFE-CFE)/DEHP composite with a DEHP mass fraction of 15wt%. This effect can be interpreted as plasticization of a polymer matrix^20^.

The melting peak enthalpy of the composites was normalized based on the amount of pure polymer and the melting peak width was taken at the half-peak height. An increase of the melting enthalpy of up to 15wt% was observed with the presence of DEHP, which implies that the addition of DEHP improved the crystallinity of the polymer to an extent of 15wt%. This can be explained by the enhanced molecular mobility of the macromolecular chains when incorporating a small quantity of plasticizer molecules.

Moreover, the composites displayed the same melting peak width of 13 °C as the pure terpolymer. The lack of dependence of the melting peak width on the DEHP content indicates that the DEHP molecules did not significantly affect the heterogeneity of the crystalline phase.

Figure 3 presents the stress versus strain curves from the measurements on the pure terpolymer and the composites with a DEHP mass fraction of 15wt%. The Young modulus of the terpolymers was extracted from the linear dependence of the stress on the strain (Fig. 3). The elasticity modulus of the neat terpolymer was calculated at E = 110 MPa. As observed in Fig. 3, a 2.5-fold decrease of the Young modulus was obtained for the P(VDF-TrFE-CFE) terpolymer filled with a 15wt% mass fraction of DEHP (Fig. 3b). Compared with other organic/inorganic electrostrictive polymer approaches, the advantage of using the DEHP plasticizer was its ability to largely maintain flexibility and toughness in the neat polymer.

Figure 4 depicts the influence of the doping agent on the low-frequency dielectric properties of the composites through dynamic dielectric spectroscopy measurements in a frequency range of 0.1 Hz to 100 Hz. The relative dielectric permittivity of the neat terpolymer remained almost constant at *ε* = 50 over the whole investigated frequency range. The doping of the fluorinated terpolymer with DEHP resulted in a significant enhancement of the low-frequency dielectric permittivity. For example at 0.1 Hz, the dielectric permittivity of the composites increased from 120 to 820 for the composites with a DEHP mass fraction between 5wt% and 15wt%. As expected, when the frequency decreased, the dielectric permittivity of the P(VDF-TrFE-CFE) composites increased. However, such an enhancement of the dielectric permittivity at low-frequency is usually accompanied by an amplification of the dielectric losses within the material.

As is illustrated in Fig. 4b, an increased dielectric loss of the materials with DEHP was observed at lower frequency range. The dramatically enhanced dielectric permittivity and the increased dielectric losses at low frequency were in accordance with the interfacial polarization according to the well-known Maxwell-Wagner-Sillars (MWS) polarization model^21^,^22^. The introduced plasticizer tended to improve the molecular mobility and thus enhance the probability of the charges becoming trapped at the heterogeneous boundaries within the polymer matrix. For all the samples, the increase of the dielectric losses at high frequency, i.e., above 10^3^ Hz, was ascribed to the dipolar orientation of the semi-crystalline terpolymer matrix^8^,^23^.

Figure 5 presents the electric field-induced longitudinal strain as a function of the applied electric field for both the pure terpolymer and its plasticizer-modified counterparts at a frequency of 0.1 Hz. As can be seen in Fig. 5a, the incorporation of DHEP largely improved the longitudinal electrostrictive strain of the terpolymer. Under an electric field of 10 V/μm, a thickness strain of *S*_33_ = 1.8% was achieved, which was 28 times higher than the strain observed in the pure terpolymer control sample. Meanwhile, the field-induced strain exhibited a quadratic dependence with the applied electric field, determined by equation (1).

It has been found that, in addition to the applied electric field, the electrostrictive strain is mainly determined by the dielectric permittivity and the Young modulus of the dielectric polymers. The greatly improved longitudinal strain at low frequency presented here was primarily caused by the aforementioned significantly increased dielectric permittivity and decreased Young modulus of the polymers with the introduction of DEHP.

However, this 28-fold increase of *S*_33_ did not match the ratio between the longitudinal electrostrictive strain constant (*M*_33_) of the filled and neat terpolymers, which was calculated from Equation 1 using the measured values of the dielectric permittivity (at a 0.1 Hz frequency) and the Young modulus. According to the theory, a twofold increase should be achieved. Consequently, another effect must be taken into account in order to precisely describe and predict the real electrostrictive strain of such materials.

Figure 5b shows the longitudinal electrostrictive strain of the P(VDF-TrFE-CFE) +15wt% DEHP composite versus the square of the electric field. A linear relationship was found to exist between the longitudinal electrostrictive strain and the square of the applied electric field under a 6.7-V/μm electric field. This phenomenon depicts a pure Maxwell strain behavior due to a constant dielectric permittivity. In other words, the longitudinal electrostrictive strain constant *M*_33_ was no longer a constant above the electric field of 6.7 V/μm, and a saturation of the electrostrictive strain occurred.

This interesting aspect can be interpreted as a saturation of the dipolar orientation. It has previously been shown that the dipolar saturation of the neat terpolymer leads to a non-quadratic dependence of the longitudinal electrostrictive strain. For the neat terpolymer^17^,^18^, the saturation electric field was nearly *E*_*sat*_ = 50 V/μm and was closely related to the dipolar orientation saturation of the dipoles of the crystalline phase within the terpolymer. Such dipolar orientation saturation is associated with a decrease of the dielectric permittivity above this electric field. For the filled terpolymer, the saturation electric field was abnormally low compared to the classical dipolar orientation. Since the increase of the dielectric permittivity is associated with charges trapped at heterogeneities - inducing large dipoles moments - the very low saturation electric field seemed related to the saturation of interfacial phenomena above a saturation electric field of 6.7 V/μm.

In order to further understand the saturation effect, Fig. 6 illustrates an estimation of the electric field saturation (*E*_*sat*_) of polymers with different mass fractions of DEHP from the strain versus electric field measurements. It has been shown that the saturation electric field of the P(VDF-TrFE-CFE) +15wt% of DEHP decreased exponentially to the amount of doping agent from *E*_*sat*_ = 50 V/μm for the neat terpolymer to *E*_*sat*_ = 6.7 V/μm . As the DEHP content was raised, the molecular mobility within the polymer increased, leading to a higher mobility of the charge carriers^22^,^24^. Above the saturation electric field of the interfacial polarization phenomena, trapped charges impeded the movement of the carriers thus causing a lower dielectric permittivity of the composite and as a result, a saturation of the electric field-induced electrostrictive strain.

Based on the physical properties of the composites, it became possible to predict the electrostrictive performances of the filled fluorinated terpolymers as a function of the applied electric field and DEHP content in order to tailor the electroactive polymer for practical applications. At a low electric field, the electrostrictive strain increased with the DEHP content until *E* = 6.7 V/μm. Above this value, the strain of the terpolymer +15wt% plasticizer leveled off whereas the one with 10% continued to increase. Thus, for practical applications, the terpolymer with 15wt% DEHP provided a more suitable fit to the requirements at a low electric field whereas in the case of higher electric fields, the 10% composite seemed more appropriate.

The dielectric breakdown strength plays a crucial role in the electrostrictive properties of dielectric polymers since it determines the practical operation electric field. During the past decades, various organic or inorganic filler-based composites have been developed to improve the electrostrictive strain of dielectric polymers^24^,^25^. However, most of these composites exhibit a large increase of the dielectric properties as well as a dramatic decrease of the dielectric breakdown strength. This is due to dispersion and surface charge accumulation between the fillers and the polymer matrix thus limiting the number of practical applications.

One of the main benefits of the approach proposed in this study is the limited effect of the doping agent on the dielectric breakdown strength of the polymer. Fig. 7 illustrates the dielectric breakdown strength of the investigated polymers using Weibull analysis. A small decrease of the dielectric voltage breakdown was achieved from *E* = 180 V/μm for the neat terpolymer to E = 150 V/μm for the terpolymer filled with 15wt% DEHP. The two curves shown in Fig. 7 indicate the top and bottom bounds of the electric field that can be applied. The composites with 5wt% and 10 wt% are between these two bounds. Moreover, the mechanism of dielectric breakdown for solid dielectrics is extremely complicated as it depends on a number of factors including molecular structure, material impurity, sample geometry, type of electrodes, ambient conditions as well as the frequency and duration of the applied electric field.

The most common breakdown mechanisms are intrinsic breakdown, thermal breakdown, electromechanical breakdown and internal disharges^26^. Compared with the same electrostrictive performances, the dielectric voltage breakdown of P(VDF-TrFE-CFE) filled with CuPc^3^ was reported to be *E*_*breakdown*_ = 13 V/μm. The solution proposed by Zhang *et al.*^3^ demonstrates the possibility to use conductive fillers to increase the dielectric permittivity by the fact that free charges not only contribute to conduction, but can also give rise to Maxwell-Wagner polarization. Nevertheless, as a drawback, such insulator-conductive composite systems tend to show losses with a percolative behavior, which may results in a dramatic increase of their conductivity for filler concentrations exceeding the percolation threshold.

Unfortunately, the maximum increase of the composite permittivity is achieved close to the percolation threshold. The parallel increases of both the dielectric permittivity and the overall losses are, of course, antagonistic. This effect seriously compromises the applicability of such types of systems as electromechanical transduction materials, due to their typically low dielectric strengths. Hence, the doping effect of (2-ethylhexyl) phthalate does not only hugely enhance the electrostrictive performances of the terpolymer but also limits its electrical and mechanical fragility, compared to conventional composites. The physical properties of this new all-organic composite make this polymer one of the most suitable dielectric materials for actuators and devices. In addition, the economic cost of CuPc is 1000-fold that of the plasticizer used, making application of the composite proposed by Zhang *et al.* very complex with regard to the socio-economic world^3^.

To further demonstrate the potential of the new all-organic composite, a specific micro-pump has been designed and tested. Indeed, micro-pumps have been studied for decades and have received much attention and interest due to a great number of applications for a broad range of modern technologies such as microfluidic techniques, biosensors, chemical analysis and delivery 27,28. Various micro-pumps have been manufactured using several actuation methods including electrostatic^29^, piezoelectric^30^, thermopneumatic^31^, shape memory alloy^32^ and electromagnetic^32^ technologies. Nonetheless, all current solutions present technological challenges, for example a sometimes insufficient displacement generated by the piezoelectric element, or SMA and thermopneumatics limited to a very low frequency range, etc^30^,^31^,^32^.

The micro-pump investigated in this study is displayed in Fig. 8 and a schematic of the nozzle diffuser architecture with the pumping chamber is given in Fig. 9. A unimorph type of diaphragm actuator consisting of electrostrictive P(VDF-TrFE-CFE) with 15wt% of DEHP was used. A 50-μm thick all-organic composite was metalized on both sides with 25 nm of a gold electrode which was patterned using a shadow mask (Cressington High Resolution Sputter Coater (208HR). The sizes of the polymer films and gold electrodes were 4.5 cm × 0.5 cm and 2.5 cm × 0.5 cm, respectively (Fig. 10b). An overlapping region of the top and bottom electrodes with a size of 0.5 cm × 0.5 cm was used as the driving membrane for micropumps, as clearly illustrated in Fig. 10c.

A circular pump chamber with a 3-mm diameter was used. This chamber dimension is typical of a fluidic pump using an electroactive material^28^. A rectangular nozzle/diffuser design with two parallel walls (top and bottom) and two converting/diverging walls (sides) was selected and is illustrated in Fig. 9. It was chosen since Olson *et al.* demonstrated that a planar diffuser can give a more compact micro-pump than a conical diffuser, due to the length for the conical diffuser with the best performance being 10%–80% longer than that of its planar counterpart.

The pumping chamber, the nozzle/diffuser and the inlet and outlet chambers were engraved in PMMA using a micro-milling machine (Roland EGX 350). The depth of the channel, nozzle and pump chamber was 100 μm. The choice of nozzle and diffuser dimensions was based on results reported in the literature^33^,^34^. The as-prepared pump without a driven membrane is illustrated in Fig. 10a (Plate A). Two through-holes were drilled at the inlet and outlet and plastic tubes with an inner diameter of 380 μm were attached to these holes from the back using epoxy glue as displayed in Fig. 8.

Figure 10d presents the assembly process of the micropump system. The metalized electrostrictive film was bonded to the engraved plate A with the electrode-overlapping region coinciding with the pumping chamber using an acrylic pressure-sensitive adhesive (3M ATG 969). The top electrode can be connected to the applied electric field through hole 2 and the bottom electrode can be connected to the ground via hole 1 (as shown in Fig. 10a). Subsequently, plate A and plate B were clamped with screws through holes 3–10. When the actuator was electrically actuated, the diaphragm bent up and down, pumping the fluid through the channel along the preferred flow direction determined by the nozzle and diffuser structures. This fabrication process is relatively low-cost and allows single-step integration of multiple active membranes into a microfluidic chip. As a promising perspective, the proposed electro-active polymer can also be integrated by thermoforming.

The implementation of the new all-organic electroactive polymer into an active pumping membrane made it possible to get a flow rates as high as 8 μl.min^−1^ with almost 250 Pa back pressure under only 15-V.μm^−1^ electric fields at 1 Hz (Fig. 11). Pumping efficiency values were measured according to the pumping characterization developed by Zhang *et al.*^35^. Similar orders of magnitude for the flow rates (25 μl.min^−1^ at 63 Hz) and the pressure (350 Pa) were achieved by Zhang *et al.*^35^ but with much a higher electric field of 90 V.μm^−1^ for the same nozzle diffuser architecture. Thanks to the considerably lower electric field applied to our new electroactive polymer, the probability of electrical failure was significantly reduced, resulting in an enhanced reliability at the integration time and an improved lifetime, as well as a lower voltage power supply.

The materials developed via a simple mixing process could potentially lead to a low-cost fabrication and the complete integration into microfluidic devices. Only one application, i.e., that of a micro-pump, has been demonstrated in this paper but other microfluidic applications such as valves could be also envisioned. This new material potential breaks the technological limitations of high voltage needed for actuation of “electronic” EAPs in microfluidic and micro-electromechanical systems (MEMS).

## Experimental section

### Sample preparation

The P(VDF-TrFE-CFE) 56.2%/36.3%/7.5% terpolymer powder was purchased from Piezotech S.A.S (Arkema Group). Films were fabricated via a solution-casting process. P(VDF-TrFE-CFE) was first dissolved in methyl ethyl ketone (MEK, Sigma Aldrich) with a polymer mass fraction of 14% and the solution was cooled at room temperature for 2 hours. Subsequently, the required quantity of bis (2-ethylhexyl) phthalate (DEHP, Sigma Aldrich) was added to the as-prepared solution and stirred for 1 hour. Finally, the mixture was cast onto a glass substrate using an Elcometer Doctor Blade film applicator and the solvent was allowed to evaporate. The films were placed in an oven at 60 °C for 12 hours to totally remove the residual solvent and were subsequently annealed at 103 °C for 1 hour to improve the crystallinity of the samples. The thickness of the final films was 50 μm. For the electrical measurements, 25-nm electrodes were gold-sputtered on each side using a Cressington High Resolution Sputter Coater (208HR).

### DSC characterization

Differential Scanning Calorimetry (DSC) measurements of the polymers with varying contents of DEHP were performed using a DSC131 evo calorimeter (Setaram Instrumentation) under a nitrogen atmosphere. For each measurement, a sample (~20 mg) was placed in a hermetic aluminum pan, heated to 160 °C where it was kept for 5 minutes, then cooled down to room temperature and kept there for another 5 minutes, and finally heated to 160 °C. Both the heating and cooling ramps were performed at 10 °C/min. Each measurement was carried out twice to ensure the reproducibility of the results. The melting enthalpy was calculated by integrating the total surface of the melting peak.

### Mechanical properties

**Figure 12:**
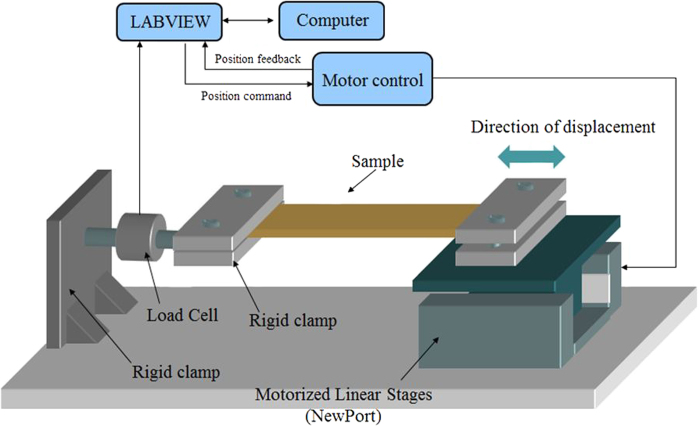
Schematic of the equipment for the stress-strain measurements.

The mechanical properties of the polymers were investigated using the system illustrated in Fig. 12. Films with a size of 4 cm × 1 cm × 50 μm were mounted in the sample holder: one side was fixed and the other side was free to move in one direction at a frequency of 100 mHz. The displacement and force were measured by a Newport table microcontroller and a force sensor, respectively, and then converted into the strain and stress of the samples. Additionally, the Young modulus of the samples was determined by the slope of the linear dependence of the stress versus strain.

### Dielectric properties and breakdown strength measurements

The dielectric properties of the polymers were determined with an SI 1296 (Solartron) impedance analyzer system in a frequency range of 0.1 Hz to 1 MHz at room temperature. The electrodes had a diameter of 2 cm, a thickness of 25 nm and were gold-sputtered on both sides for the dielectric measurements

By definition, the dielectric strength is the maximum voltage required to produce a dielectric breakdown through the material and is expressed in volts per unit thickness. In other words, the dielectric strength is a measure of the electrical strength of a material as an insulator and the higher its value, the better is the insulating quality. Three basic procedures can be used to determine the dielectric strength including a short-time method, a slow rate-of-rise method, and a step-by-step method^26^. All of them depend on the same basic set-up consisting of the test specimen placed between two electrodes in air or oil.

In this study, the short-time method was employed. For this, the voltage was applied across the two electrodes and increased at a uniform rate (0.5 kV/s) from zero until dielectric breakdown. Breakdown can be observed when an electrical burn-through punctures the sample, or decomposition occurs in the specimen. The rate of the increase in voltage is determined by the time it takes the sample to reach dielectric breakdown. In the preparation of test specimens, great care was taken to get the surfaces adjacent to the electrodes parallel and as plane and smooth as the material would permit.

The dielectric strength of an insulating material varies with the thickness of the test specimen. Therefore, tests on specimens of different thicknesses are not comparable. In this paper, dielectric breakdown strength characterizations were carried out on 30-μm thick films metalized with round electrodes that were 8 mm in diameter. Since the thickness of the specimens was low, the test could be carried out in air. In fact, specimens over 1 mm thick are typically tested in oil to decrease the chance of flashover before breakdown.

For each type of composite, 30 samples were used to determine the probability of voltage breakdown. A tip connected to the high voltage amplifier (Treck 10/10) was used to apply the voltage to the samples (Treck 10/10). The current and voltage measurements of the high-voltage amplifier were recorded on an oscilloscope. The breakdown field was identified when the voltage source switched to a current source supplying a short-circuit current set to 10 mA. The maximum voltage supplied was also recorded using a voltmeter connected to the source via a HV probe and was considered as the breakdown voltage.

### Electromechanical characterization

**Figure 13:**
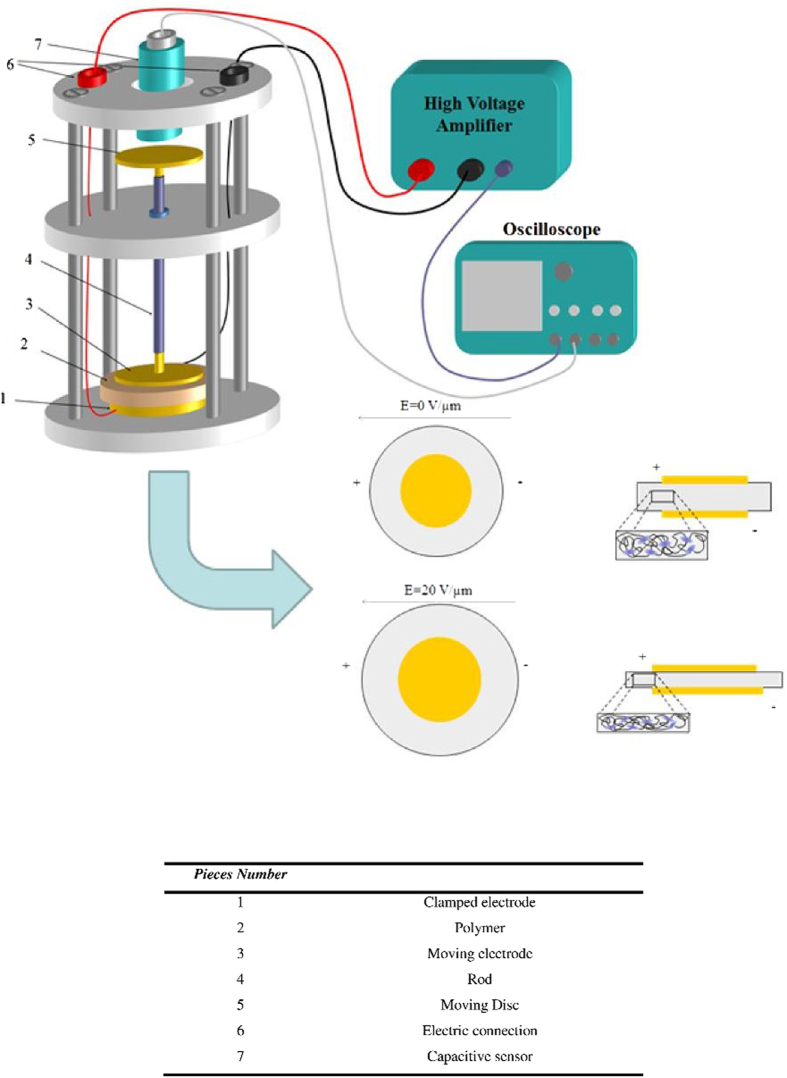
Principle of the electromechanical characterization setup using a displacement sensor. The operational principle of a conventional electrostrictive polymer before and after actuation. Developing an electrostatic stress between oppositely charged electrodes squeezes the electrostrictive polymer, thereby causing a lateral displacement of the specimen.

Figure 13 shows the setup for measuring the strain versus electric field in compression mode. The strain was determined with the help of a non-contact capacitive measurement sensor (FOGALE MC 940) with a precision on the order of 10 nm. Since the P(VDF-TrFE-CFE) sample is elastically much softer than ceramics and the samples were formed into very thin films, great care had to be taken during the strain measurements to ensure the accuracy of the data when an electric field was applied. For such soft films, flexure motion and mechanical clamping of a sample are two major causes of error in the strain measurements.

The film samples were placed on horizontal stainless steel discs (20 mm in diameter) in order to avoid measuring a parasitic flexure motion, and a second brass disc was positioned on top of the films, thus rendering it possible to apply a bipolar electric field. The total weight of parts 3, 4 and 5 shown in Fig. 12 was 5 g (equivalent to 156 Pa), which was a suitable small stress ensuring that clamping of the sample could be avoided. The sample was subjected to an electric field with the help of a waveform generator (Agilent 33220A) for which the output was amplified through a high-voltage amplifier (model 10/10, Trek Inc.). The displacement and voltage were monitored with the help of an oscilloscope (Agilent - DSO7034A). The strain was deduced by dividing the displacement by the initial thickness.

## Conclusion

This paper reports on a DEHP-plasticized P(VDF-TrFE-CFE) composite (polymer alloy) with very attractive electrostrictive properties. The insertion of a small quantity of plasticizer molecules resulted in the improvement of the molecular mobility of the macromolecules and a decrease of the Young modulus and melting temperature. Meanwhile, the increase in molecular mobility also enabled the trapping of charges at the boundaries of heterogeneities within the polymer leading to a greatly enhanced dielectric permittivity and increased dielectric losses within a low frequency range. As a result, a significant improvement of the electrostrictive longitudinal strain at low frequency was observed, 28-fold that in the pure terpolymer (control sample) at a 10 V/μm applied field.

For polymers with a higher content of DEHP, saturation of the electrostrictive strain was obtained earlier due to the saturation of the dipolar orientation inside the polymer. The relationship between the strain saturation and DEHP contents was revealed. Furthermore, compared with traditional composites, the DEHP-modified polymers exhibited a slightly decreased dielectric breakdown strength, which was considered a great advantage for practical actuator applications. The effectiveness of using the developed material for a micro-pump application was demonstrated. The proposed all-organic composites with excellent electrostrictive properties are promising for developing high-performance actuators with low stimulation electrical voltages.

## Additional Information

**How to cite this article**: Le, M. Q. *et al.* All-organic electrostrictive polymer composites with low driving electrical voltages for micro-fluidic pump applications. *Sci. Rep.* **5**, 11814; doi: 10.1038/srep11814 (2015).

## Figures and Tables

**Figure 1:**
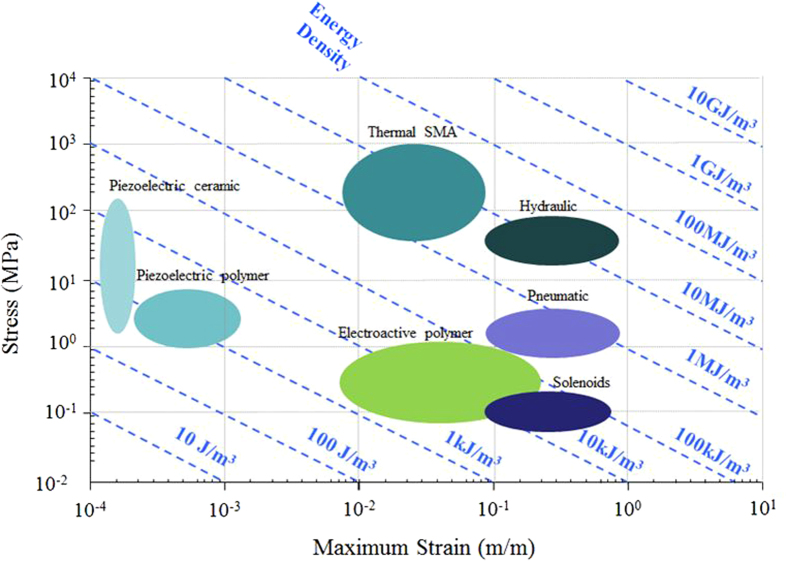
Typical stress and strain characteristics of actuation technologies.

**Figure 2:**
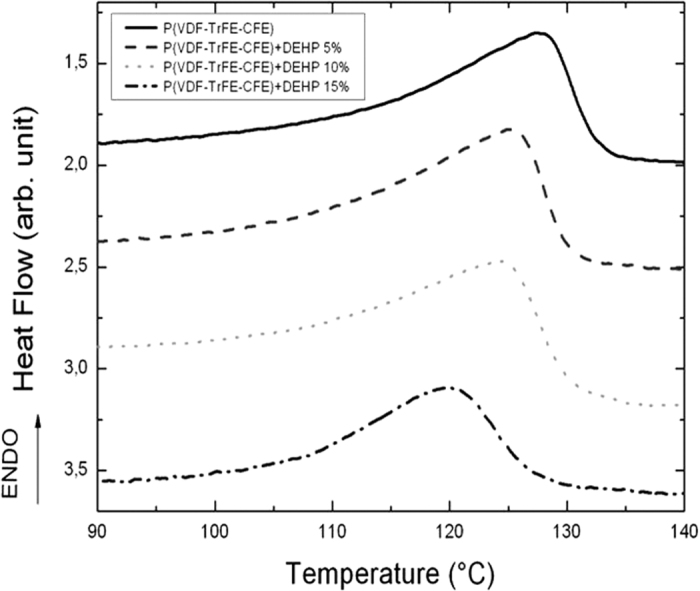
DSC thermograms for P (VDF-TrFE-CFE) polymers with different mass fractions of DEHP.

**Figure 3:**
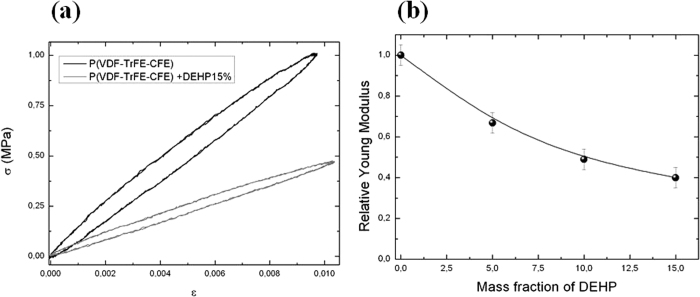
(**a**) Longitudinal stress-strain curves and (**b**) relative Young modulus (normalized to the Young modulus of the neat terpolymer) for P(VDF-TrFE-CFE) polymers with different mass fractions of DEHP.

**Figure 4:**
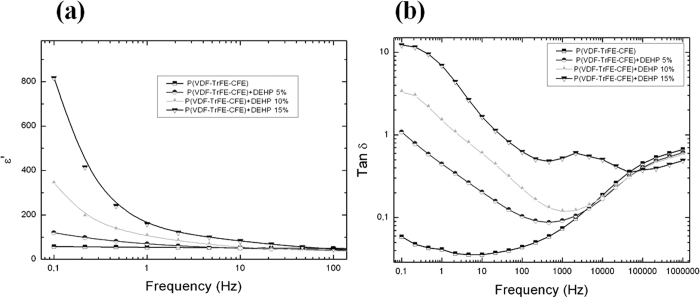
(**a**) Dielectric permittivity and (**b**) dielectric loss as functions of frequency at room temperature for the P(VDF-TrFE-CFE) polymers with different mass fractions of DEHP.

**Figure 5:**
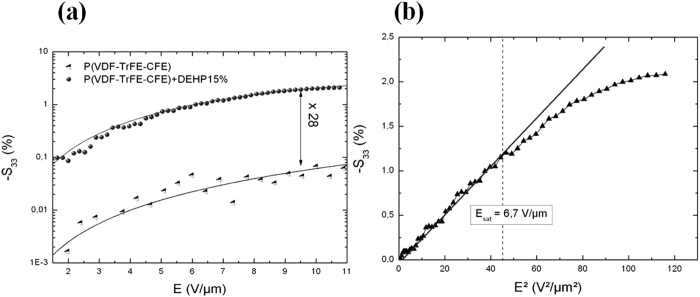
(**a**) Longitudinal electrostrictive strain versus the applied electric field for P(VDF-TrFE-CFE) and its composites with a DEHP mass fraction of 15wt% (scatter) (the lines serve as guides to the eye). (**b**) Longitudinal electrostrictive strain versus the squared applied electric field for the terpolymer and its composites with a DEHP mass fraction of 15wt% at 0.1 Hz. The solid line represents the theoretical values described with the Maxwell effect using eq. (1).

**Figure 6:**
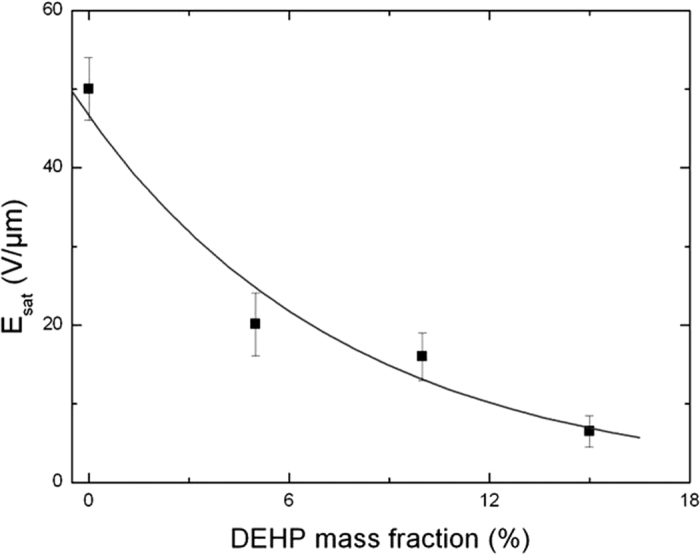
Evolution of the saturation electric field for the P (VDF-TrFE-CFE) polymers with different mass fractions of DEHP.

**Figure 7:**
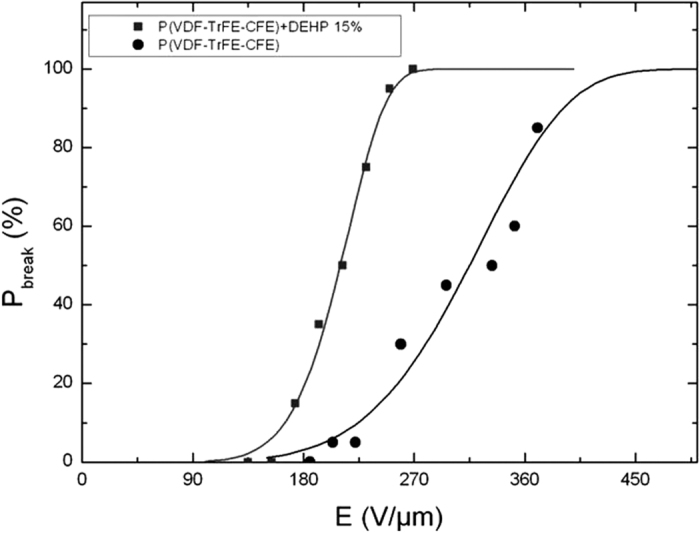


**Figure 8:**
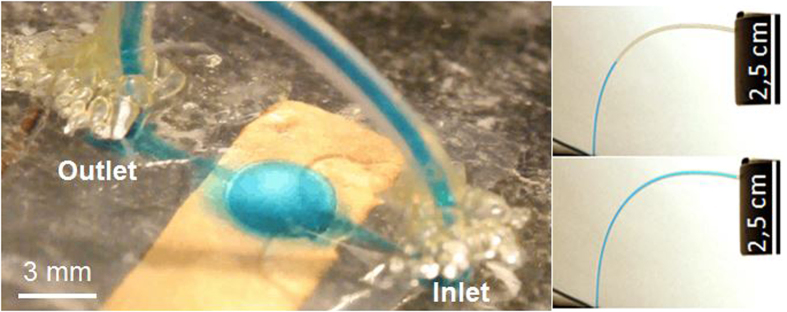
Photographs of the micro-pumping system (left) and outlet tube (right) before and after actuation of the pumping membrane.

**Figure 9:**
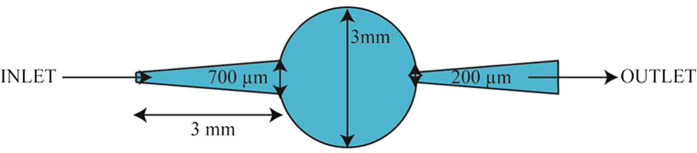
Schematic of the nozzle/diffuser architecture.

**Figure 10:**
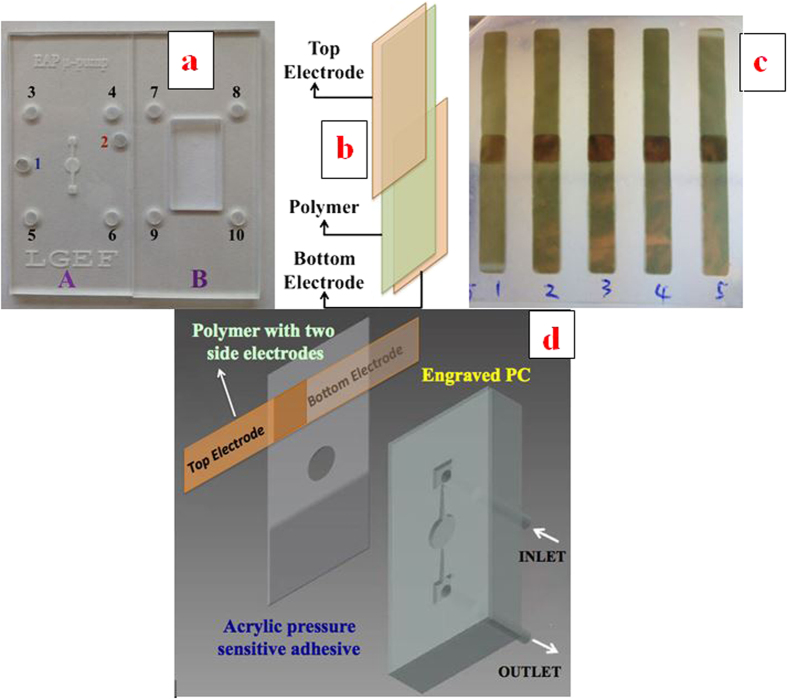


**Figure 11:**
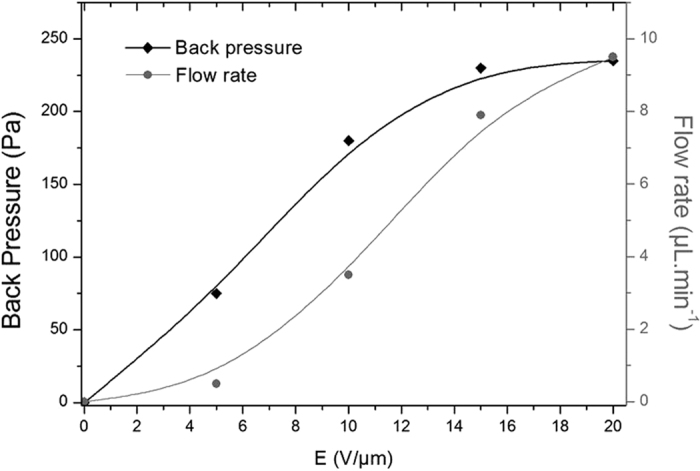
Flow rate and back pressure function of the electric field at 1 Hz (the lines serve as guides to the eye).

**Table 1:** Comparison of electromechanical properties of ferroelectric relaxor polymers.

Materials	Y(Gpa)	S_m_ (%)	YS_m_^2^/2 (J/cm^3^)	E(V/μm)
Irradiated P(VDF-TrFE) [11]	0.38	4	0.3	150
P(VDF-TrFE-CFE)/CuPc [12]	0.75	1.91	0.13	13
P(VDF-TrFE-CFE) [13]	0.3	4.5	1.1	130
P(VDF-TrFE-CTFE) [14]	0.4	4	0.32	150
